# Buffalo College of Rational Medicine

**Published:** 1879-05

**Authors:** 


### Buffalo College of Rational Medicine.

The key note of an enormous horn, "*Educated in both Schools*," broken, or about to be broken!! At the small end of the horn, is being *screached* out upon the similia similibus curantur platform, (stolen from those who built it)—educated in the Buffalo Medical College of *Ra-tion-al* (?) Medicine. The only reply yet heard at the opposite end of this gigantic horn is the astonished response, *Ra-tion-al!!!*

